# The C4 protein from the geminivirus *Tomato yellow leaf curl virus* confers drought tolerance in Arabidopsis through an ABA‐independent mechanism

**DOI:** 10.1111/pbi.13280

**Published:** 2019-11-23

**Authors:** Miguel Corrales‐Gutierrez, Laura Medina‐Puche, Yanling Yu, Liping Wang, Xue Ding, Ana P. Luna, Eduardo R. Bejarano, Araceli G. Castillo, Rosa Lozano‐Duran

**Affiliations:** ^1^ Shanghai Center for Plant Stress Biology Chinese Academy of Sciences Shanghai China; ^2^ Center for Excellence in Molecular Plant Science Chinese Academy of Sciences Shanghai China; ^3^ Instituto de Hortofruticultura Subtropical y Mediterránea “La Mayora” (IHSM‐UMA‐CSIC) Area de Genética Facultad de Ciencias Universidad de Málaga Málaga Spain; ^4^ University of the Chinese Academy of Sciences Beijing China

**Keywords:** geminivirus, drought, C4, TYLCV, tomato, ABA

Drought causes important declines in crop yield, negatively impacting plant growth, physiology and reproduction. Plants have adopted a wide range of strategies to confront the negative effects of drought, including increasing water uptake by optimizing the root system, closing stomata to limit the water loss caused by transpiration, accumulating osmoprotectants, or producing the hormone abscisic acid (ABA). This intricate scenario makes drought resistance a highly complex trait, with polygenic nature, low heritability, and vastly influenced by genotype‐environment interactions (Fang and Xiong, 2014).

A growing body of evidence indicates that viral infections can result in improved plant tolerance to abiotic stresses (see, among others: Aguilar *et al.*, 2017; Anfoka *et al.*, 2016; Westwood *et al.*, 2012; Xu *et al.*, 2008), raising the tantalizing idea that the identification of the molecular mechanisms underlying these viral effects could unlock yet‐unknown stress tolerance strategies and pave the way for the generation of stress‐resilient plants. Geminiviruses are viruses with small circular, single‐stranded DNA genomes that infect a broad range of plants. The geminivirus *Tomato yellow leaf curl virus* (TYLCV) is a main causal agent of Tomato yellow leaf curl disease (TYLCD), one of the most devastating viral diseases affecting tomato crops in tropical and temperate areas worldwide. Interestingly, infection by TYLCV has been shown to alleviate heat stress responses in tomato (reviewed in Gorovits *et al.* 2019). Here, we show that infection by TYLCV enhances drought tolerance in tomato and *Nicotiana benthamiana* and that the virus‐encoded protein C4 is the viral determinant conferring drought tolerance in Arabidopsis through an ABA‐independent mechanism.

In order to determine whether the infection by TYLCV could affect drought tolerance in its natural host, tomato, as well as in the model *Solanaceae* species *N. benthamiana*, we agroinoculated plants with a TYLCV infectious clone and subjected half of the infected plants to drought treatment at 21 days post‐inoculation. Interestingly, TYLCV‐infected plants from both species wilted more slowly after the drought treatment and displayed milder drought‐related symptoms, suggesting that the presence of TYLCV promotes drought tolerance (Figure 1a,b).

**Figure 1:**
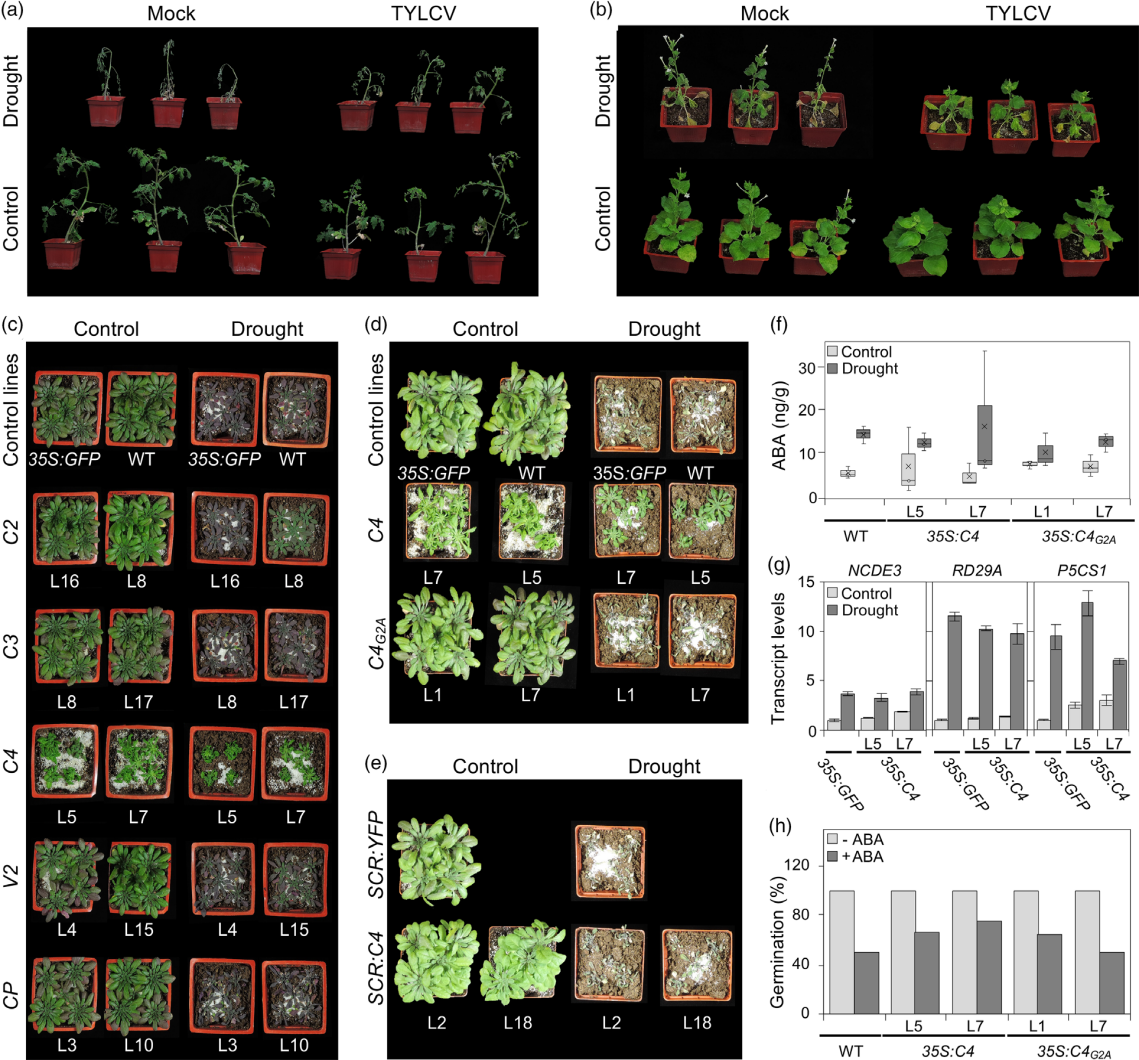
C4 from TYLCV confers drought tolerance in *A. thaliana*. Tomato (cv Money maker) (a) *or N. benthamiana* (b) plants infected with TYLCV or mock‐inoculated in drought (after reducing the amount of water supply to 5 mL per day for one week) or control conditions at 28 days post‐inoculation (dpi). (c) Six‐week‐old transgenic Arabidopsis plants expressing TYLCV genes under the 35S promoter and control plants (WT and transgenic *35S:GFP*) after withholding water for 31 days. (d) Seven‐week‐old transgenic Arabidopsis plants expressing wild‐type C4 or the non‐myristoylable mutant C4_G2A_ under the 35S promoter or (e) wild‐type C4 under the *SCR* promoter after withholding water for 39 days. Three independent biological replicates were performed with similar results; results from one biological replicate are shown in (a), (b), (c) and (d). (f) ABA content in 4‐week‐old transgenic Arabidopsis plants *35S:C4* and *35S:C4_G2A_* after withholding water for 13 days. Values represent the mean ± SD of three plants. (g) Relative transcript levels of ABA‐responsive genes (*NCDE3*, *P5CS1* and *RD29A*) in transgenic *35S:C4* or control (*35S:GFP*) plants after withholding water for 30 days. Five independent plants for each line were pooled, and values were relativized to those in *35S:GFP* plants in control conditions; *ACT2* was used as normalizer. Values represent the mean ± SD of three technical replicates. (h) Germination rate of transgenic Arabidopsis *35S:C4* and *35S:C4_G2A_* seeds sown in the absence (‐ABA) or the presence of exogenous ABA (0.3 μM, +ABA) for 6 days; wild‐type (WT) plants are used as control. 24 seeds were used per line; two biological replicates were performed with similar results; results from one replicate are shown.

In order to find out whether one of the virus‐encoded proteins is sufficient to confer the observed enhanced drought tolerance, we subjected transgenic Arabidopsis lines constitutively expressing the viral genes (C2, C3, C4, V2 and CP) from a 35S promoter to drought treatment and evaluated their performance. Constitutive expression of Rep renders plants non‐viable; hence, this protein was not included in this study. Drought did not affect expression of the transgenes (data not shown). As shown in Figure 1c, among the viral proteins tested, only C4 had an impact on the tolerance of Arabidopsis to drought: *35S:C4* transgenic plants stayed green and turgid following water deprivation, while transgenic plants expressing other viral proteins or control plants wilted and eventually died.

C4 localizes both at the plasma membrane and in chloroplasts (Rosas‐Díaz *et al.*, 2018). Given this double subcellular localization pattern, we wondered whether C4 is promoting drought tolerance through its specific activity at one of these two locations. In order to answer this question, we tested the drought tolerance of transgenic Arabidopsis plants expressing the mutated C4 version, C4_G2A_, which accumulates exclusively in chloroplasts (Rosas‐Diaz *et al.* 2018). Despite showing transgene expression levels similar to those observed in *35S:C4* plants, *35S:C4_G2A_* plants displayed sensitivity to drought comparable to that of wild‐type plants (Figure 1d), indicating that chloroplastic C4 is not sufficient to confer drought tolerance and suggesting that its plasma membrane localization is required for this effect.

Transgenic expression of C4 in Arabidopsis has been recently shown to interfere with xylem patterning in the root (Fan *et al.*, 2019). With the purpose to determine whether the effect of C4 on drought tolerance could be uncoupled from its impact on xylem patterning, we evaluated the drought tolerance of *SCR:C4* transgenic Arabidopsis plants, which express C4 under the endodermis‐specific *SCARECROW* (*SCR*) promoter but display xylem patterning defects similar to those observed in *35S:C4* plants (Fan *et al.*, 2019). As shown in Figure 1e, *SCR:C4* plants did not show enhanced drought tolerance, indicating that the effects of C4 on xylem patterning and drought tolerance are independent and that the latter requires expression of C4 outside of the *SCR* expression domain.

Since ABA is the main hormone regulating drought responses in plants, we decided to assess the effect of C4 on ABA accumulation and responses by (i) quantifying ABA accumulation and (ii) measuring the expression of ABA‐responsive genes in basal and drought conditions in wild‐type, *35S:C4* and *35S:C4_G2A_* plants and (iii) evaluating the ability of *35S:C4* and *35S:C4_G2A_* plants to respond to exogenously applied ABA, compared with the wild‐type control. Interestingly, C4‐expressing plants did not differ from wild‐type plants in any of the readouts measured (Figure 1f, g, h, respectively). Taken together, these results indicate that C4 does not significantly affect the ABA pathway and therefore must be promoting drought tolerance in an ABA‐independent manner.

Considering the current prospects of growth in world population, increasing crop yield is a cornerstone to guarantee food security. In a context of climate change, boosting agricultural productivity will necessarily entail generating not only high‐yielding, but also stress‐resistant crops. While traditional breeding has provided valuable solutions in the past, the current scenario makes it essential to explore alternative approaches, which may allow to narrow the temporal gap between identification of a beneficial trait and its implementation in the field. The study of extremophile species has been suggested as having the potential to unlock novel sources of resistance (Zhang *et al.*, 2018). Similarly, unravelling the physiological and molecular mechanisms underlying the beneficial effects of virus infection on abiotic stress tolerance may uncover new approaches to generate stress tolerance, informing strategies to speed up the generation of crop varieties with increased resilience to abiotic stresses.

Our results demonstrate that the virus‐encoded plasma membrane‐localized C4 protein is sufficient to confer drought resistance to Arabidopsis plants (Figure 1c). At the plasma membrane, C4 interacts with the receptor‐like kinases BAM1 and BAM2; however, BAM1, which is inhibited by C4 (Fan *et al.*, 2019; Rosas‐Díaz *et al.*, 2018), plays a positive role in the response to drought (Takahashi *et al.*, 2018). More importantly, the effect of C4 on drought tolerance seems independent of ABA (Figure 1f, g, h). Taken together, these facts support the idea that C4 confers drought tolerance through a BAM1‐independent, yet‐to‐be‐identified mechanism.

Viruses have been shown to enhance drought tolerance through the promotion of salicylic acid (SA) signalling (Aguilar *et al.*, 2017). C4‐expressing plants, however, do not overaccumulate SA and do not display increased expression of SA marker genes (data not shown), indicating that the events underlying the effect of C4 on drought stress tolerance are different to the ones previously described for other viruses (Aguilar *et al.*, 2017; Westwood *et al.*, 2012; Xu *et al.*, 2008).

In summary, our findings demonstrate that C4 from TYLCV increases plant drought tolerance in an ABA‐independent manner through a mechanism that relies on the presence of this protein at the plasma membrane. Considering the dramatic increase in survival upon water withholding mediated by C4 and the fact that this effect is independent of well‐known hormonal pathways linked to drought stress, we believe this discovery to entail great biotechnological potential. Future efforts will focus on unravelling the molecular events underpinning the C4‐triggered enhanced tolerance to limited water availability: the identification of the viral drought tolerance‐promoting strategy could open up new avenues to explore in the engineering of the highly sought‐after drought‐tolerant crops.

## Author contributions

ERB, AGC, and RL‐D conceived and supervised the project; MC‐G, LM‐P, and YY generated and analyzed data; LP and XD generated materials; APL, ACG, and RL‐D wrote the manuscript with input from all the authors.

## Conflict of interest

The authors declare no conflict of interest.
